# 

**Published:** 1857-06

**Authors:** 


					﻿Receipts on Account of Medical Journal, A// J- C. Hughes.
Dr. Adamson,V V $2 00
Atkinson, '3 00
Brady,	3	00
Boon Harry, 2 00
Beerbower J, 2 00
Bailey M, 2 00
Catamole, 2 00
Cousins,	6	00
Cochran, 2 00
Crawford, 2 00
Cobb G M, 2 00
Dozz FA, 2 00
Davidson J W, 2 00
Davis EC, 2 00
Ellison J J, 2 00
Evans,	2	00
Fry,	4	00
Fitch,	2	00
Gutch Wm. ) 2 00
Holiday G R, 2 00
Dr. Hart, J G, $4 00
Hall R W, 2 00
Hood,	2	00
Henderson, 2 00
Hensay J C, 3 00
King,	2	00
Johnson P D, 3 00
Johnson, 2 00
Keables, 2 00
Kellogg, A II, 2 00
Lowrey,	2	00
Lewis D B, 2 00
Moor Robert, 2 00
McRaynolds J, 2 00
Morse L I),	2	00
Norris,	5	00
O’Brien,	2	00
Potter,	2	(Ml
Russell VV A J, 2 00
Ross,	2	0O
Dr. Rand FB, $2 00
Rogers,	1	00
ScolesHJ, 2 50
Skinner,	2	OO
Scott, James 2 (Ml
Stillman C B, 2 OO
Swartz G II, 2 00
SevillM, 2 00
Taylor SW, 200
Timmonds, 5 00
Taylor C. 2 00
Turner,	2	00
Taylor,	2	00
Trimble John, 2 00
Van Patten, J’ 2 00
Williamson, 2 OH
Warford, 2 00
Wood,	2	OH
Whitted,	2	OO
Shedd George, 4 00
X5T We have just received the first three numbers öf a new Jour-
nal on Dentistry, “ L’ Revue Mensuelle De La Chirurgie Et De
La Prothese Dentaires ”—-edited by M. M. Fowler & Preterre,
American Dentists. Published in Paris. We welcome it upon our
•exchange list.
New Books.
We have received the following books from the publishers, Blanch-
ard & Lea, which will be fully noticed in our next number :
Taylor’s Medical Jurisprudence ; Allen’s Dissector’s Guido ;■
Bownan’s Practical Chemistry ; Introduction to Practical Pharmacy,
Parrish ; Churchill on Children—second American Edition ; and
Bartlett on the Fevers of the United States—fourth and Revised
Edition.
Receipts on Journal.—In order to prevent mistakes and save the
time required in answering letters, relative to moneys received upon,
payment of Journal, we will insert the name, with amount received,,
upon the cover of each issue.
Necrology.—Died, in London, on the 24th of December last, Dr
John A. Paris, President of the College of Physicians.
Died, in Wheeling, Va., in May last, Dr. Hullion, a Dental Sur-
geon, of extensive reputation in his profession.
Notice of Cards-—We would call the attention of the profession to
■the numerous ad ver ii cements which will be found in an appended
form and upon the cover of this work. A number of these deserve
especial notice, which we shall attend to in our next issue. ■,
JJ≤rDr. Timmonds, of Knoxville, Iowa, while on his way home
from this city a few weeks since, was stabbed by a fellow traveler,
the knife entering the thorax and penetrating the left lung. Al-
though at first life was despaired of by his friends, we are happy to
state he is convalescent.
				

